# Beyond Weight Loss: Establishing a Postbariatric Surgery Patient Support Group—What Do Patients Want?

**DOI:** 10.1155/2018/8419120

**Published:** 2018-02-14

**Authors:** Saira Hameed, Victoria Salem, Tricia M. Tan, Alma Collins, Krishna Shah, Samantha Scholtz, Ahmed R. Ahmed, Harvinder Chahal

**Affiliations:** ^1^Imperial Weight Centre, Imperial College Healthcare NHS Trust, St Mary's Hospital, Praed Street, London W2 1NY, UK; ^2^Section of Investigative Medicine, Division of Diabetes, Endocrinology and Metabolism, Imperial College London, Hammersmith Campus, Hammersmith Hospital, 6th Floor Commonwealth Building, Du Cane Road, London W12 0NN, UK; ^3^Imperial College School of Medicine, Imperial College London, South Kensington Campus, London SW7 2AZ, UK; ^4^Department of Surgery and Cancer, Imperial College London, St Mary's Campus, Praed Street, London W2 1NY, UK

## Abstract

**Purpose:**

There are limited resources for long-term specialist follow-up after bariatric surgery. In selected centres, patients can access a postoperative support group, but there is no clear evidence to guide their delivery.

**Materials and Methods:**

A retrospective study of bariatric surgery patients (*n* = 152) who had been discharged from specialist follow-up (mean time since surgery 5.5 years), covering weight history, physical and psychosocial comorbidities, and the need for a postoperative bariatric support group.

**Results:**

Fifty-eight percent wanted a postbariatric surgery patient support group. This was not associated with operation type or the amount of weight lost or regained. However, those who wanted a support group were significantly more likely to be struggling to keep the weight off, to be unhappy with the way they look, or to be experiencing difficulties returning to work.

**Conclusions:**

These data point to an unmet patient requirement for a postoperative support group that is independent of weight loss success. More research is required to ascertain how such a group should be delivered, but our data would suggest that supporting patients with weight loss maintenance, body image, and return to work is an important part of postoperative care, and these needs extend well beyond the immediate period of specialist follow-up.

## 1. Introduction

The global prevalence of obesity is increasing. Overall, 13% of the world's adult population is obese, a prevalence that has more than doubled since 1980 and is projected to continue to rise [1]. Bariatric surgery is the only intervention that has been shown to result in sustained weight loss of a magnitude to confer cardiovascular and metabolic benefits [2, 3]. The provision and duration of postoperative follow-up in most surgical units are limited by finite resources; bariatric surgery in itself is not a panacea, and a deterioration in mental health or new physical morbidities can appear many years after surgery, including weight regain [3, 4] and psychological ill health such as depression and poor body image [5].

A patient support group is an alternative model to individual follow-up that is more resource efficient whilst allowing extended contact with health care professionals [6]. Following bariatric surgery, there is a general agreement that such groups are beneficial to long-term outcomes—including weight loss and emotional well-being [7–9]. The importance of postoperative support groups has been underscored in the United States, where their provision is a requisite for conferring the status of a “Bariatric Center of Excellence” [10].

Scant research exists as to the design and delivery of a postoperative support group. Furthermore, there is paucity of information about what patients themselves would like from such a group. Given that weight loss and small magnitude weight regain from the postoperative nadir are commonly used markers of surgical success [11], we hypothesised that those patients who had experienced poor weight loss or marked weight regain would be the patients who expressed the need for extended postoperative support.

The objectives of this study were to investigate whether patients with less postoperative weight loss and/or weight regain would be more likely to request a postoperative support group compared with patients who had achieved the expected postoperative weight loss and/or weight maintenance; to identify any factors in common between patients requiring a postoperative support group; and to identify the needs of patients requesting a postoperative support group.

Data were collected using a questionnaire sent to patients who had undergone Roux-en-Y gastric bypass, gastric sleeve, or gastric banding within our unit and who had since been discharged from specialist follow-up. The questionnaire was designed to assimilate Patient Reported Outcome Measures (PROMs). PROMs are widely used to capture an individual patient's experience of their situation which might be different from that of their caregiver [12]. PROM data allows for the patient's perspective to inform how health care is delivered and, in the case of this study, to ascertain what patients themselves want from a postoperative support group.

## 2. Materials and Methods

We have conducted a retrospective study using a detailed questionnaire that asks patients about weight outcomes and other factors including physical and mental health which determine the requirement for a postoperative support group. The patients included in this study had undergone Roux-en-Y gastric bypass (RYGB) or sleeve gastrectomy or gastric band at Imperial College Healthcare NHS Trust (ICHNT) from 2007 to 2013. The routine follow-up programme within our unit at each postoperative time point is as follows: 10 days with a clinical nurse specialist (CNS); 3 months with a dietician; 6 months, 1 year, and 2 years with a CNS. Clinical review and laboratory tests at each of these appointments will determine the need for review by other members of the multidisciplinary team, for example, surgeon, physician, psychologist, and psychiatrist. After 2 years, patients are discharged back to their primary care doctor who can re-refer the patient to our unit if needed. Patients were included if they had been discharged from specialist follow-up in our unit.

A manual search of our bariatric surgery database yielded the contact and clinical details of 923 potential participants at the time of the survey. A questionnaire requesting demographic data (the provision of patient-identifiable information was optional) and pre- and postoperative variables (including self-reported weight history and physical and psychosocial comorbidities) was posted to patients. Questions were asked about specific PROMS relating to their physical outcomes, postoperative complications, postoperative experiences, and their requirement for a postoperative support group. Patients were asked to delete as appropriate a “Yes/No” list of postoperative physical complications: diarrhoea or constipation that was not there before surgery; excess or loose skin; gallstones; hernias; issues surrounding pregnancy; others (please specify); low blood sugar levels after eating; ulcers in the stomach or gut; and vitamin deficiencies or malnutrition. Patients were also asked to delete a “Yes/No” list of sixteen questions relating to their postoperative experiences: I have not lost as much weight as I had hoped; I lost a lot of weight at first but have now regained some/all; I have lost too much weight; I am finding it difficult to maintain the original weight loss; I am actively dieting to keep the weight off; I am struggling to keep the weight off; the health problems that I had before surgery did not improve as much as I had hoped; I am taking more medications than I had hoped to be needing after the surgery; my relationship with food has not improved; I miss food/eating; I have looked to other things in my life to replace what food used to provide, for example, alcohol; I am not happy with the way I look; I do not spend as much time as I would like taking care of myself; I have found returning to work difficult; the surgery has affected my relationship with my partner/spouse; and the surgery has affected my relationship with other people, for example, friends/family.

The questionnaire culminated with detailed questions about the need and preferred format for a postoperative bariatric support group. Patients who answered yes when asked if they would be interested in a postoperative support group were asked their views on how this should be delivered with the following options offered: frequency of support group sessions (once a week/once a fortnight/once a month/every three months); session duration (30 minutes/1 hour/1.5 hours/2 hours); preferred time of day (daytime (0900–1700)/evening (1800–2000)); preferred venue (in the hospital where the operation was performed/community or church hall/other (please specify)); ideal patient group size (less than 10 people/10–15 people/15–20 people/more than 20 people); other sources of postoperative support that might be of use (contact by text message, e-mail bulletins, or other (please specify)) seminars/talks, social media groups). Patients who answered “no” to the need for a postoperative support group were asked why they had come to this decision. The following options were given: I am not comfortable taking part in a group; I feel my life has moved on and I do not want to think about my surgery anymore; I get a lot of support from other health care providers, for example, my general practitioner; I have nothing that I want to talk about; I live a long way away; Other (please specify); the cost of the travel; the inconvenience to my daily routine, for example, finding someone to look after the kids.

A prepaid, addressed envelope was provided for return of the questionnaire. Participants were given 105 days between January and April 2016 to respond.

Data analysis was initially conducted on the entire group of responders. Patients were then subclassified in a binary fashion as to whether they did or did not want a support group. Responses were compared between the two groups, with particular attention to “no weight regain” or “weight regain.” We applied a cutoff of 15% weight regain between the preoperative and nadir postoperative weight (i.e., a 15% regain of the maximum postoperative weight loss) to define weight-regainers (as previously described) [13]. The percent overall weight loss was calculated as (preoperative–current weight)/preoperative weight. Finally, a range of categorical data and free text responses were thematically analysed regarding what the patients wanted from a support group.

Continuous data are presented as mean ± standard error of the mean (SEM) and categorical data using counts and percentages. Differences in starting weight and weight loss between groups were compared. Chi-square tests and *t*-tests were used to determine the relationship of proportions of categorical variables between those who wanted a support group with those who did not. *p* values of ≤0.05 were considered statistically significant. Statistical analysis was performed using GraphPad Prism 6.

## 3. Results

There was a completed questionnaire return rate of 16.6% (*n* = 152). An additional 35 questionnaires (3.8%) were returned as “not known at this address.” Of those responding to the survey, 88 respondents (58%) stated that they would be interested in a postoperative support group. A summary of our respondents' characteristics stratified by whether they did or did not want a support group is summarised in Table 1.

Overall, 77% of responders were female, which is representative of our entire bariatric surgical cohort, and this proportion was similar amongst those who requested a support group (77% women) and those who did not (75% women). The average age of our survey responders at the time of surgery was 45.7 years, and the mean time since surgery was 5.5 years. Twenty-four patients who responded to our survey had received a gastric band (15.8%), 95 patients a RYGB (62.5%), and 33 patients had undergone a sleeve gastrectomy (21.7%). There was no significant difference in the distribution of surgery types between those who wanted and those who did not want a support group (see column 3, Table 1).

Respondents were categorised according to National Economic Census (NEC) social class. NEC social class 8 (unemployed) was the largest cohort to be represented (28.9%), although there was no significant difference in the rate of requests for postoperative support across the entire NEC scale.

Since these patients had been discharged from specialist follow-up, we examined whether two or more visits to their general practitioner with a postoperative issue was a characteristic of patients who required a support group. Thirty-eight out of the 88 patients (43.2%) who wanted a postoperative support group had seen their general practitioner on more than two occasions regarding their bariatric procedure. Twenty-five out of the 64 patients (39%) who did not want a support group had seen their general practitioner twice or more times regarding a postoperative issue. Our results show that people who requested a support group were not more likely to have visited their general practitioner with a bariatric-related problem (*p*=0.67) with the majority of all responders having visited their general practitioner with a bariatric problem on less than two occasions since surgery.

We initially hypothesised that those patients who had achieved a lesser degree of weight loss would be more likely to require a postoperative support group. The average weight loss in the group that had undergone banding was 20.1% compared to 21.7% in the sleeve gastrectomy group and 42.3% in the RYGB group. Across the entire cohort of survey responders, the average percentage total weight loss at the time of taking the survey compared with preoperative weight was 28.2% which is in line with other published series [14]. The percentage weight loss was not different between those who wanted a support group and those who did not with a mean weight loss of 30.9% in the group who wanted a support group compared with 27.7% weight loss amongst those who did not want a support group (*p*=*ns*). Furthermore, there was no difference in the preoperative body weight between the two groups (138.3 ± 2.7 kg in those who wanted a support group compared with 142.9 ± 3.7 kg in those who did not).

The questionnaire asked questions about weight regain. The responses were based on self-reported measures of preoperative weight, nadir postoperative weight, and current body weight. Forty-four responders (28.8%) were classed as weight regainers (i.e., by definition >15% weight regain from nadir). The magnitude of weight regain ranged from 15.4% to 103.8%. There was no difference in percentage weight regain between any of the surgical procedures.

Percentage weight regain was not different between those who wanted a support group and those who did not, with weight regain averaging 13.8% in those who requested a support group compared with 11.3% in those who did not (*p*=*ns*).

Since weight loss, weight regain, and surgery type were not determinants of the patient requirement for a postoperative support group, we sought to establish which features of their bariatric experience did relate to such a request. We examined whether the presence of a range of postoperative morbidities related to interest in a support group (Table 2). No single postoperative complication was significantly more common amongst the cohort who request a support group, although there was a trend towards a higher incidence of dumping syndrome and loose skin in those who did express an interest in postoperative support.

Patients were then presented with 16 independent questions requiring a “yes/no” response about their postoperative experience. These are listed in Table 3. We performed a chi-squared test to see whether the proportion of positive responses differed according to whether patients wanted or did not want a support group. There were three contingency tables that were associated with a significantly higher “yes” response amongst those who expressed a desire for a support group: these were, “I am struggling to keep the weight off” (*p*=0.02); “I am not happy with the way I look” (*p*=0.04); and “I have found returning to work difficult” (*p*=0.03).

Finally, we examined what the respondents who did express an interest in a support group wanted from it. Questions were asked with “delete as appropriate” answer options given as well as the opportunity for a further free text response. These questions related to the frequency of the support group, the duration of a support group session, the venue, the ideal group size, and ideas for ancillary means of support such as e-mail bulletins and social media groups.

Of the 88 respondents who requested ongoing postsurgical support, 46% requested one meeting a month, whilst 38% wanted a support group meeting every 3 months. Most respondents wanted the session to last for one hour, and they were split equally between whether they preferred a daytime or evening slot, although 3 out of every 4 respondents wanted it to occur in the hospital where they had their operation (rather than a community-based setting).

The most common request (from 65% respondents) was for educational sessions in the form of seminars and talks across a range of subject matters related to weight loss surgery, but a similar proportion was also interested in e-mail bulletins. Twenty-nine percent requested a private social media group in order to communicate with fellow patients. Patients requested support in three areas: firstly, they wanted formal education delivered by experts about obesity, nutrition, exercise, and the weight loss surgery itself; secondly, they were looking for moral support from others who had experienced a similar journey; and thirdly, they expressed a desire to be able to keep in contact with professionals with a specialist interest in obesity should the need arise.

For those who did not want a postsurgery support group, the reasons for this could also be broadly divided into three categories: firstly, there were those who were happy with the results and did not feel that they needed any more help (25%), a further 15% said that they were not comfortable with a public arena and the remainder said that the inconvenience (cost of travel or other constraints) meant that they were unlikely to find a support group useful.

## 4. Conclusion

This detailed survey of our bariatric surgery patients who have been discharged from specialist follow-up reveals that the majority of patients would like access to a support group.

These PROM data are important because they provide patient-centred information beyond health professional-led measures of success after bariatric surgery such as the degree of weight loss or percentage of weight regain, neither of which determined the demand for a postoperative support group. It is important to note that due to the constraints of the data collected, the starting body weight does not represent preoperative body mass index. However, there was a similar amount of weight loss and weight regain between those who did and did not want a postoperative support group. Furthermore, surgery type, gender, social class, and the presence of postoperative complications did not influence the interest in a postoperative support group.

Three markers were significantly different between those who did and those who did not want a support group. The first was that, despite achieving a similar degree of weight loss and weight regain as patients who did not want a support group, those who wanted one were “struggling to keep the weight off.” In some cases, this was associated with active dieting. The mechanisms by which bariatric surgery results in weight loss are myriad and include changes in postprandial gut hormone responses affecting perceived satiety [15], changes in taste preference [16, 17], and the perceived reward response to high sugar, high-fat foods [18, 19]. Although beyond the scope of this work, it may be that some or all of these postoperative changes are less profound in certain patients, who therefore experience a greater challenge achieving a given degree of weight loss. This would point to the need for enhanced support for these patients that extends beyond the usual model of postoperative nutritional advice.

Poor body image was also more common amongst those patients who wanted a support group. Despite losing as much weight as those who did not want a support group, these patients remained unhappy with their appearance. Although the (self-reported) weight loss achieved by our cohort of survey responders is in line with other surgical series [14], this may reflect a dissonance between caregiver and patient expectations of the weight loss achievable with bariatric surgery [20]. There was also a hint from the data presented here that the occurrence of loose skin following profound weight loss is a major limitation in patients' perception of success [21]. It is widely recognised that there is a high prevalence of body image dysphoria amongst obese patients [22] and although physical change may have been achieved, this might not have occurred in tandem with the appropriate psychological change.

Of particular socioeconomic interest here is that difficulties with return to work were prevalent and significantly more likely to be expressed by patients who wanted a support group. The data collected in this survey were not designed to allow us to interrogate the specific issues related to transition back to work and it follows that further research is needed on this front. However, the economic and health benefits of a successful return to gainful employment when they are achieved are clear [23].

The current UK model is that two years after bariatric surgery, patients are discharged back to primary care for ongoing management [24]. We follow this guideline unless there are active ongoing postoperative complications. Although limited by the potential reporting bias of a retrospective, self-reported study, the information returned to us by our bariatric patients suggests that our concepts of surgical success are not always aligned with those of our patients who feel the need for continued physical and psychosocial support.

At present, the majority of late postoperative issues will be managed by general practitioners despite the identification of barriers to the comprehensive management of these complex patients in a primary care setting [25, 26]. Nevertheless, this model of care within the specialist unit for two years after surgery followed by primary care-managed shared care is described in the recently published UK commissioning guidance for weight assessment and management in adults and children with severe complex obesity [27]. Such a model provides a balance between the needs of a specialist patient group that often has complex medical needs and the reality of finite secondary care resources. Given that two years after operation, the majority of patients will be looked after in primary care, support groups can provide an efficient link with ongoing specialist support, compared to the traditional model of clinic attendance.

There is limited evidence in the literature regarding optimal professional and peer support following bariatric surgery, and their measures of success are often (inconclusively) measured by successful weight loss [8, 28–31]. In addition, our work suggests that patients would find technological interfaces such as e-mail contact and social media to be useful components of postoperative support. Interestingly, apps for use on mobile devices are increasingly available for perioperative bariatric support [32].

Further studies are required before firm conclusions can be drawn; however, this study begins to provide a flavour of what our patients themselves want from such a service and what psychosocial as well as physical elements of postoperative “success” need to be considered.

## Figures and Tables

**Table 1:** Respondents were stratified according to whether they wanted (*n* = 88) or did not want (*n* = 64) a postoperative support group. Exact *n* numbers, mean, and standard deviations (SD) are given. There was no significant difference in age, time since surgery, preoperative weight, or average percentage total weight loss with surgery when these two groups were compared using a Student's unpaired *t*-test. Chi-square proportion testing did not reveal any significant differences between the two groups in terms of gender or social class distribution, average weight regain, or operation type. *p* values for each of these statistical comparisons are stated.

	Want a support group (*n* = 88)	Do not want a support group (*n* = 64)	Comparison *p* value
% female	77% (*n* = 68)	77% (*n* = 49)	*p*=0.92
Average age (years)	50.4 (SD 10.0 yrs)	56.4 (SD 10.1 yrs)	*p*=0.13
Average time since surgery (years)	6.1 (SD 2.2 yrs)	5 (SD 7.4 yrs)	*p*=0.95
*Operation type*			
Gastric band	12.50% (*n* = 11)	20.30% (*n* = 13)	*p*=0.19
RYGB	69.30% (*n* = 61)	53.10% (*n* = 35)	*p*=0.06
Sleeve gastrectomy	18.20% (*n* = 16)	26.60% (*n* = 17)	*p*=0.22
Average preoperative weight	138.3 kg (SD 25.1 kg)	142.9 kg (SD 28.4 kg)	*p*=0.30
Average percentage total weight loss	30.90% (SD 13.24)	27.70% (SD 13.32)	*p*=0.56
Average weight regain (proportion of max weight loss)	13.80%	11.32%	*p*=0.29
*Weight regain*			
>15% of maximal loss	31.8% (*n* = 28)	25.0% (*n* = 16)	*p*=0.36
*NEC (UK National Economic Census)*			
(1)	5.7% (*n* = 5)	9.4% (*n* = 6)	*p*=0.39
(2)	17.1% (*n* = 15)	15.6% (*n* = 10)	*p*=0.82
(3)	17.1% (*n* = 15)	7.8% (*n* = 5)	*p*=0.10
(4)	2.3% (*n* = 2)	4.7% (*n* = 3)	*p*=0.41
(5)	3.4% (*n* = 3)	1.6% (*n* = 1)	*p*=0.48
(6)	5.7% (*n* = 5)	10.9% (*n* = 7)	*p*=0.24
(7)	10.2% (*n* = 9)	7.8% (*n* = 5)	*p*=0.61
(8)	28.4% (*n* = 25)	29.7% (*n* = 19)	*p*=0.86
R (retired)	9.1% (*n* = 8)	12.5% (*n* = 8)	*p*=0.50

**Table 2:** Respondents were stratified according to whether they wanted (*n* = 88) or did not want (*n* = 64) a postoperative support group. This was analysed against the self-report of a range of well-described postsurgical complications. Contingency analysis (chi-squared tests) revealed that the frequency of each complication asked about was not statistically higher in the cohort who requested postoperative support.

Postoperative condition	Want a support group (*n* = 88)	Do not want a support group (*n* = 64)	Chi-squared *p* value
	% (number) with the condition	% (number) with the condition	
Dumping syndrome	60.2% (53)	48.4% (31)	0.151
Diarrhoea/constipation	45.5% (40)	40.3% (26)	0.432
Loose skin	70.5% (62)	61.3% (39)	0.155
Gallstones	9.1% (8)	9.67% (62)	0.952
Hernia	12.5% (11)	11.3% (7)	0.691
Pregnancy issues	4.5% (4)	4.84% (3)	0.967
Postprandial hypoglycaemia	15.9% (14)	11.3% (7)	0.381
Peptic ulcers	3.4% (3)	1.61% (1)	0.483
Vitamin deficiencies/malnutrition	53.4% (47)	53.2% (34)	0.822

**Table 3:** A list of sixteen questions (yes/no) were asked of our cohort. We had a total of 154 responders, divided into two groups: those who stated they wanted a postoperative support group (*n* = 88) and those who did not (*n* = 64). This table tallies the number of positive (YES) responses to each question by the group. We performed contingency analysis (chi-squared test) for each question by group (*p* values for each test are given in the final column). This revealed that the *n* = 88 responders who wanted the support group were statistically more likely than those who were not interested in a bariatric support group to answer “yes” to question numbers 6, 12, and 14. This did not survive multiple comparisons (the Bonferroni adjustment).

		Number who agreed		Chi-square test *p* value
		Want support group (*n* = 88)	Do not want support group (*n* = 64)	
Q1	I have not lost as much weight as I had hoped	**22**	**40**	0.171
Q2	I had lost a lot of weight at first, but have now regained some/all	**31**	**52**	0.265
Q3	I have lost too much weight	**1**	**0**	0.239
Q4	I am finding it difficult to maintain the original weight loss	**22**	**37**	0.338
Q5	I am actively dieting to keep the weight off	**13**	**26**	0.198
Q6	I am struggling to keep the weight off	**18**	**43**	0.018
Q7	The health problems that I had before the surgery did not improve as much as I had hoped	**16**	**18**	0.507
Q8	I am taking more medications that I had hoped to be needing after surgery	**10**	**16**	0.679
Q9	My relationship with food has not improved	**21**	**31**	0.757
Q10	I miss food/eating	**10**	**14**	0.962
Q11	I have looked to other things in my life to replace what food used to provide, e.g., alcohol	**5**	**11**	0.353
Q12	I am not happy with the way I look	**15**	**36**	0.412
Q13	I do not spend as much time as I would like taking care of myself	**10**	**13**	0.885
Q14	I have found returning to work difficult	**0**	**6**	0.033
Q15	The surgery has negatively affected my relationship with my partner/spouse	**5**	**10**	0.469
Q16	The surgery has negatively affected my relationship with other people, e.g., friends/family	**4**	**14**	0.069
